# Dietary Effects on Pain Symptoms in Patients with Fibromyalgia Syndrome: Systematic Review and Future Directions

**DOI:** 10.3390/nu15030716

**Published:** 2023-01-31

**Authors:** Emma K. Maddox, Shawn C. Massoni, Cara M. Hoffart, Yumie Takata

**Affiliations:** 1 BioHealth Science Program, College of Science, Oregon State University, Corvallis, OR 97331, USA; 2 Department of Microbiology, College of Science, Oregon State University, Corvallis, OR 97331, USA; 3 Department of Pediatrics, Division of Rheumatology, Children’s Mercy Kansas City, Kansas City, MO 64108, USA; 4 School of Medicine, University of Missouri-Kansas City, Kansas City, MO 64108, USA; 5 School of Biological and Population Health Sciences, College of Public Health and Human Sciences, Oregon State University, Corvallis, OR 97331, USA

**Keywords:** fibromyalgia syndrome, fibromyalgia patients, diet, pain symptoms

## Abstract

Fibromyalgia syndrome (FMS) is recognized for its difficulty to diagnose and its subjective symptomatology. There is neither a known cure nor a recommended therapeutic diet to aid in the multidisciplinary treatment. We conducted a systematic review to investigate if diets can improve pain symptoms of fibromyalgia. Through the PubMed search in March 2022, 126 abstracts were identified. We included both intervention and observational studies of diets and pain symptoms among patients with FMS. After screening titles, abstracts, and full-texts, 12 studies, including 11 intervention and one observational study, were selected. These studies included 546 participants and investigated plant-based diets (*n* = 3), anti-inflammatory diets (*n* = 1), gluten-free diets (*n* = 2), and elimination/restrictive diets (*n* = 6). These studies assessed pain symptoms through visual analogue scale for pain, fibromyalgia impact questionnaire/revised fibromyalgia impact questionnaire, tender point count, pain pressure threshold, and/or total myalgic score. Nine studies, including all three plant-based diet studies, reported statistically significant beneficial effects of their respective diets on pain symptom measurements. Given the small sample size and short intervention duration of the included studies, limited evidence currently exists to recommend any specific diet to patients with FMS. Further research is warranted to clarify specific diets to recommend and explore their potential mechanisms.

## 1. Introduction

Fibromyalgia is a complex chronic syndrome that is largely characterized by subjective symptoms. There is no known cause or cure although symptoms may be managed via multimodal treatment strategies [1]. The most frequent symptoms of fibromyalgia syndrome (FMS) include widespread pain and tenderness, fatigue, stiffness, headaches, issues with sleep and cognitive functions. Among the less common symptoms are digestive problems, tingling or numbness of extremities, and face and jaw pain [1]. This chronic syndrome affects around 2–3% of the world’s population [2]. Fibromyalgia predominately affects women to men at a ratio of 3:1 [2]. Some individuals experience a rapid onset of FMS, which often occurs after an illness or traumatic incident, whereas others may not have a triggering event [1].

The diagnostic process identifying this disease can be difficult, due to widespread, subjective symptoms and the lack of a known clear etiology [2]. Theories explaining underlying causes are evolving and recently fibromyalgia has been described as a central sensitization disorder, in that those with FMS have a heightened sensitivity to pain due to improper pain signal processing by the central nervous system [1]. Fibromyalgia may be caused by environmental and genetic factors operating in tandem [1]. It is hypothesized that the central nervous system and the peripheral nervous system are involved in the pathological mechanisms of FMS [2]. Given the likely complex biopsychosocial pathology of this condition, a comprehensive approach, including physical and psychological interventions, are the ideal treatment of FMS. Fibromyalgia patients may suffer from whole body pain, fatigue, stiffness, hypersensitivity to external stimuli, and autonomic disturbances [2]. Additionally, fibromyalgia can have a substantial impact on mental health; patients can develop anxiety, depression, and post-traumatic stress disorder [2]. Potential cognitive difficulties include memory deficits, concentration difficulties, and sleep disturbances [2]. Consequently, there are multiple treatment modalities required due to the complexity of the syndrome [1].

Currently, exercise is the most recommended treatment method as it has been shown to reduce pain symptoms and fatigue; however, there is currently no known diet or vitamin supplementation recommended for the treatment of FMS [2]. Dietary interventions are a tool used for the treatment of many diseases, due to healthy diets improving physical fitness, mental health, and cognitive abilities [3]. For example, plant-based diets have been used to treat hypertension as they significantly lower systolic and diastolic blood pressure [4]. However, the current status of the evidence on dietary influences on FMS is not known. Therefore, we investigated if diet affects pain symptoms for individuals with fibromyalgia through a systematic literature review of published studies and summarized future research directions.

## 2. Materials and Methods

A systematic literature review was conducted using the PubMed online database to identify published studies. The following search terms were applied for (all fields): diet AND fibromyalgia. This search was conducted with filters for studies conducted in humans and articles written in English and with no limits on publication years. The final search was completed on 7 March 2022, and a total of 126 abstracts were retrieved and reviewed independently by two authors (E.K.M. and Y.T.), who discussed discrepancies when they occurred and brought them to consensus. The full-text article of a study was not acquired if the abstract or title met at least one of the following exclusion criteria: (1) not including fibromyalgia participants; (2) not using visual analogue scale for pain (VAS), fibromyalgia impact questionnaire/revised fibromyalgia impact questionnaire (FIQ/FIQR), tender point count (TPC), pain pressure threshold (PPT), or total myalgic score (TMS) measurements; (3) not testing diet as intervention or exposure variable; and (4) not being an original research article such as a review or commentary. The studies that met at least one of the exclusion criteria were separated from the remaining abstracts. The full-text article was obtained for all the remaining abstracts that did not meet the exclusion criteria for the abstract or title to be further reviewed. The articles were excluded if they met at least one of the following exclusion criteria: (1) having an overlap of study participants between studies; (2) not including fibromyalgia participants; (3) not using VAS, TPC, PPT, FIQ/FIQR, or TMS measurements; (4) not testing diet as intervention or exposure variable; and (5) not being an original research article such as a review or commentary. The protocol of this systematic review was not registered.

Each study was cited, and the following information was compiled: first author; year of publication; country; study design; calendar year the study was conducted; number of participants; age; sex; race or ethnicity; length of intervention; presence of control group; and study results. Not all the information was included in the published article of all 12 studies. In these instances, the studies were examined for references cited, searching for related articles about the study through PubMed, and/or contacting the corresponding author to obtain missing information if possible. Two authors (E.K.M. and Y.T.) extracted the information mentioned above from each study independently. Inconsistencies were discussed and brought to consensus.

An outcome variable of this literature review was pain symptoms. Each study used the evaluation procedures including the VAS, TPC, PPT, TMS, and/or FIQ/FIQR. The FIQ allows patients to report the severity of their pain, fatigue, stiffness, anxiety, depression, morning tiredness, physical impairment, mood, and ability to go to and do work. The FIQR allows patients to report the severity of how FMS interferes with their function, overall impact, and symptoms, including pain and tenderness. The FIQ and FIQR values of each subsection were added and converted to fit into a 0–100 scoring range, with the higher values indicating more severe FMS symptoms. The VAS is used to measure the intensity of pain commonly on a 0–100 mm length line, where the 0 mm region is absence of pain, and the 100 mm region is the maximum pain imaginable [5]. The TPC was an essential measurement of the 1990 American College of Rheumatology (ACR) criteria but had since been phased out for the 2010 ACR criteria. A tender point is indicated by the patient feeling pain from a 4-kg palpitation on one of the 18 designated sites [6]. The PPT is commonly performed on the 18 tender sites and measures the amount of pressure over a given area, specifically the point where non-painful pressure changes into painful pressure. The TMS is the PPT score over the tender points. For all pain symptom measures, the average values and standard deviations for each group (e.g., intervention and control groups) at each time point (e.g., the baseline and the end) for intervention studies and, for a cross-sectional study, the average values of pain symptom measures by quartiles of dietary inflammatory index (DII) scores at one time point were extracted. When studies reported average values differently (e.g., average changes during the intervention, instead of the average values at the end), we estimated the average values using the available information.

The included studies were analyzed based on their participant inclusion and exclusion criteria; recruitment method; method of questionnaire/pain symptom measurement; type of diet that was investigated; how diet was administered and monitored; length of intervention; presence of control group; study results; study’s strengths and limitations; and authors’ conclusion. For the study results, we assessed the certainty of each study’s results based on the reported statistical significance (*p*-values < 0.05). Risk of bias of each study was assessed through the National Heart, Lung, and Blood Institute (NHLBI) study quality assessment tools [7] and independently by two authors (E.K.M. and Y.T.). Discrepancies were discussed and brought to consensus.

## 3. Results

**Figure 1:**
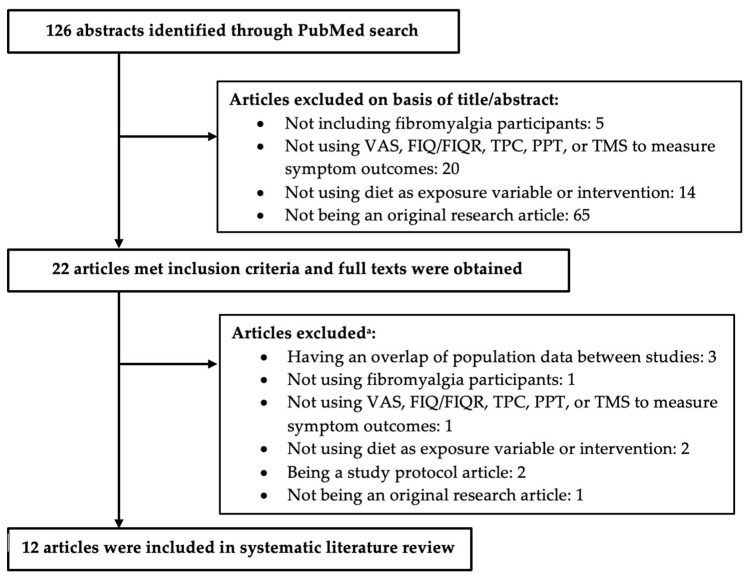
Flow chart of the systematic literature review. Abbreviations used: visual analogue scale for pain (VAS), fibromyalgia impact questionnaire/revised fibromyalgia impact questionnaire (FIQ/FIQR), tender point count (TPC), pain pressure threshold (PPT), or total myalgic score (TMS). **^a^** The following articles were excluded due to having an overlap of study participants between studies [8,9,10]; not including fibromyalgia participants [11]; not using VAS, TPC, PPT, FIQ/FIQR, or TMS measurements [12]; not testing diet as intervention or exposure variable [13,14]; and not being an original research article such as a review or commentary [15].

This review examined 126 abstracts that were published before March 2022 using the previously stated search terms. The abstracts and titles of these 126 articles were reviewed (Figure 1) based on the exclusion criteria; 20 articles were selected for full-text review. From the 20 full-text articles, 12 articles met the final inclusion criteria and were included in our literature review.

The 12 studies were published spanning from 2000 to 2020 and were primarily intervention studies except for one study [16], which was an observational, cross-sectional study (Table 1). These studies took place in seven different countries, four in Spain, three in the United States, and one in each of the following countries, Bangladesh, Finland, Portugal, Italy and Egypt. The sample sizes of all 12 articles were relatively small, with a minimum of 7 participants [17] and a maximum of 95 participants [16], totaling 546 participants. Five of the reviewed studies included solely female participants [16,17,18,19,20], and six studies included female participants as the majority with less than 25% male participants [21,22,23,24,25,26]. Hänninen et al. did not report the sex, race, or ethnicity of their participants. Only two studies [17,18] reported race or ethnicity and included all white participants. All 12 studies used ACR criteria for diagnostic inclusion criteria, specifically nine used ACR 1990 criteria, but three studies [17,19,24] used ACR 2010 criteria. Multiple studies had participants with comorbidities, as it is common for FMS patients to have additional illnesses. Marum et al. [19] reported that 88% of their participants had a gastrointestinal disorder and 60% had food intolerance in addition to their FMS diagnosis. Three other studies had at least one comorbidity as additional inclusion criteria [17,23,24]. In terms of study quality, three studies rated as good quality [24,25,26], five as fair quality [16,17,19,21,23] and four as poor quality [18,20,22,27], respectively.

The twelve studies were grouped into four categories based on the type of diet. The first category, plant-based diets, consisted of three studies: a vegetarian diet [21], a raw vegetarian diet [22] and a living food diet, which is defined as an uncooked vegan diet [27]. The second category, gluten-free diets, contained two studies [17,24]. The next category is an anti-inflammatory diet, which was solely comprised of one study [16]. The final category consisted of the remaining six studies that implemented elimination/restrictive diets. Marum et al. [19] introduced a diet that eliminated foods high in fermentable oligo-, di- or mono-saccharides and polyols (FODMAPs). Lamb et al. [18] implemented a diet that eliminated simple sugars, artificial colorings, flavorings, and sweeteners; caffeinated beverages; grains with gluten; eggs and dairy products; allergenic foods; and foods high in arachidonic acid. Both Vellisca and Holton studies [20,23] implemented excitotoxin elimination diets. Vellisca and Latorre [20] focused on eliminating monosodium glutamate (MSG) and aspartame. Holton et al. [23] had participants eliminate both MSG and aspartame but included a list of other excitotoxic food additives to avoid as well. Pagliai et al. [25] implemented the Khorasan Wheat Replacement diet where wheat products made with Khorasan wheat were provided to the intervention group and the same products made with regular wheat were provided to the control group. Another study implemented an energy-restrictive diet [26].

Among the three plant-based dietary intervention studies, in the Azad study [21], the vegetarian diet group’s mean VAS score statistically significantly decreased from 5.7 at baseline to 5.0 at the end of the six-week intervention, which was a smaller change than the control group which decreased from 6.2 to 2.3. However, this control group was given amitriptyline to help with insomnia (Table 2). The mean TPC had a statistically significant decrease in the control group (from 16.1 to 6.4), but not in the vegetarian diet group (from 15.7 to 14.7) (Table 3). In the Donaldson study [22], the mean FIQ decreased from 51.4 at baseline to 27.6 at the end of seven months of living food diet intervention with a statistical significance. In the Hänninen study [27], the vegan diet intervention resulted in a statistically significant decrease in their mean VAS scores over three months (specific mean values not reported but presented in graphs).

An anti-inflammatory diet was only investigated in one observational, cross-sectional study. Correa-Rodríguez et al. [16] completed a 24-h dietary recall and one-time measurement of FIQR, VAS, and PPT. The participants were categorized into quartiles based on their dietary inflammatory index (DII) scores where lower scores represented an anti-inflammatory diet and higher scores represented a pro-inflammatory diet. All locations of the PPT measurements were significantly associated with a lower DII quartile (Table 4). There was no significant association of lower FIQR and VAS scores with DII scores.

As the last category, six studies used an elimination/restrictive dietary intervention. Marum et al. [19] used a low FODMAP diet and found that both the mean FIQR and VAS scores significantly decreased from 61.6 at baseline to 47.9 at the end of the four-week trial, and from 6.6 to 4.9, respectively. Lamb et al. [18] implemented a hypoallergenic, modified elimination diet with phytonutrient-rich medical food followed by a control period of a USDA food pyramid diet with a rice protein powder supplement for four weeks each in women with FMS. They reported a mean FIQ score of 46.3 at baseline, 43.6 at the end of the USDA food pyramid diet and 36.6 at the end of the intervention period. This decrease in the FIQ score was not statistically significant, although the mean FIQ sub-section pain score had a statistically significant decrease (5.5 at baseline, 5.94 at the end of USDA control diet and 4.92 at the end of the intervention period). No statistically significant differences were reported for various bodily areas of PPT measurements over time. Vellisca and Latorre [20] implemented a diet that eliminated MSG and aspartame for three months. Their VAS score scale was not conventional as it only ranged from 0–7. Although there were some improvements in pain in both the control and intervention groups over three months, neither group achieved statistical significance. Holton et al. [23] implemented a diet that eliminated additive excitotoxins from their patients with FMS and IBS diagnoses. Eight of the participants reduced their tender points to less than 11 after four weeks of the intervention. In the Holton study [23], their average FIQ, VAS (0–20 score scale), TPC, and TMS scores decreased by 22.2, 5.4, 2.5, and 9.5 after four weeks, respectively. Senna’s [26] energy-restricted diet intervention for six months resulted in a statistically significant decrease in FIQR. Although no baseline measure was taken, TPC was lower in the intervention group than the control group at the end of the intervention. For PPT, five out of nine areas assessed had a statistically significantly lower PPT in the intervention than the control diet groups. Besides the FMS pain measures, the intervention group experienced more weight loss than the control group. In the Pagliai study [25], only FIQR was measured and FIQR scores at baseline did not differ between the Khorasan Wheat Replacement diet and control diet groups. After eight weeks of the intervention, the participants who received the Khorasan wheat products had a statistically significantly lower average FIQR score.

## 4. Discussion

We conducted this systematic literature review to investigate if diet has the potential to provide symptom relief for those with fibromyalgia based on published studies. Among the 12 studies reviewed, eight intervention trials reported statistically significant improvements in at least one of the pain measurements as a result of the intervention and one cross-sectional study observed an inverse association between DII score and pain measurements. By diet categories, all of the plant-based diet and anti-inflammatory diet studies found statistically significant improvement in pain measurements based on FIQ, VAS, TPC or PPT [16,21,22,27]. Inconsistent results were reported for gluten-free and elimination/restrictive diets. Only one out of the two gluten-free diet studies [17] reported statistically significant improvements in pain measurements including FIQ, VAS and TPC. Four out of the six elimination/restrictive diets reported statistically significant pain improvements in FIQR/FIQ, VAS, TPC, TMS or PPT [19,23,25,26]. 

Overall, it is encouraging that the majority of the studies included in this review reported a pain improvement in at least one of the measurements. Although we were able to cover a variety of diet types, specific diet types that are effective to alleviate pain symptoms among patients with FMS are not clear. In our systematic review, plant-based diets reported the most consistent results [21,22,27] and the only anti-inflammatory diet study also reported significant pain improvement [16]. Both diets were similar in terms of high consumption of vegetables, fruits, vegetable/olive oils and nuts, and low consumption of red meats. Furthermore, some elimination diets in our review share commonalities. Food additives were avoided in Holton, Lamb and Vellisca studies [18,20,23]. As food additives are contained in processed foods, the living food diet in the Donaldson study would also restrict the consumption of food additives [22]. Among these four studies, only two reported a statistically significant improvement in pain symptoms with the intervention diet [22,23]. Hence, the effectiveness of eliminating food additives on pain symptoms among patients with FMS is currently inconclusive and needs to be clarified in future studies. With regards to gluten-free diets, only one of the two trials reported a significant pain improvement [17]. The diet used in the Lamb study is an elimination/detoxification diet that also excluded gluten from the diet and no significant difference in the pain symptom changes between the intervention and control diets were reported [18]. Potential reasons for these inconsistent results are that gluten-free diets had no specific guidance on the amount of fruit, vegetables, nuts and red meats allowable, and they did not restrict consumption of processed foods high in food additives.

One potential mechanism that may explain our finding of more consistent results from plant-based and anti-inflammatory diets than gluten-free diets is weight loss. Among patients with FMS, weight loss is associated with improved pain symptoms [28]. Plant-based and vegetarian diets are generally associated with lower body weight [29]. For the Hänninen study [27], weight loss in the vegan diet group was reported [8], although body weights were not reported in the other two plant-based diet studies. In contrast, a gluten-free diet may not necessarily result in weight loss; instead, a healthful weight gain was reported among celiac patients following a gluten-free diet as it helped to alleviate malabsorption/digestive issues [30]. In the Slim study, the control diet, not the gluten-free diet, group experienced significant weight loss. Among elimination/restrictive diet studies, the Senna study among obese patients with FMS [26] reported both pain improvement and weight loss as a result of the energy-restricted diet intervention. In the Marum study [19], the low FODMAP diet group had significant weight loss [9]. Given that only four of the 12 studies reported the participants’ weight or BMI changes during the diet intervention period, future studies are needed to report their weight changes to help elucidate this potential mechanism.

Besides body weight, other potential and hypothesized mechanisms are through decreased inflammation and decreased activation of neurotransmitters in central sensitization. In the Senna study, the intervention group had more pain improvements and weight loss as well as lower concentration of inflammatory markers (i.e., *C*-reactive protein and interleukin-6) than the control group. This study highlights the effects of body weight on pain symptoms, potentially mediated through inflammatory pathways. The finding from the Correa-Rodríguez study [16] also lends support for decreased inflammation as a potential mechanism, given that participants whose diets were characterized as higher anti-inflammatory dietary potentials had lower scores of pain measurements. The cross-sectional nature of this study limits us to consider the weight change over time, which was not reported, as an additional mechanism to explain their finding. Future studies including biomarkers to assess inflammation and central sensitization would also help to elucidate potential mechanisms. Additionally, diets other than those included in this review may also result in pain improvement. For instance, low-carbohydrate and ketogenic diets were recently hypothesized to alleviate chronic pain, also through inflammatory and nervous system pathways [31], and other diets also need to be investigated in future studies of pain symptoms among FMS patients.

In addition, future studies should take into consideration the variability of adherence to the respective diet being studied. The majority of studies in our review did not report the adherence rate to the diet regimen and a few studies noted challenges in adhering to the diet regimen experienced by study participants [21,22]. Moreover, all 12 studies included outpatient participants who may have experienced additional difficulties regarding adherence as they were having to make a major dietary change upon enrolling in a dietary intervention trial on top of managing one or more chronic conditions and completing tasks for their daily life. To increase their adherence, a meal plan service that is vetted by researchers could help alleviate the challenges that participants may experience. The majority of the studies reviewed did not report providing participants with adequate resources and introductory sessions with dietitians. As an exception, the Holton study provided detailed instructions for the dietary regimen (e.g., a list of excitotoxic food additives to avoid), individual professional dietary counseling sessions and food diaries to complete three days a week, all of which might have contributed to a significant pain improvement in a short period of four weeks [23]. Hence, future studies may also consider providing detailed instructions and support for participants to follow the dietary regimen as part of the intervention trial.

One strength of this systematic literature review is that the 12 studies took place over seven different countries and four continents with varying cultures, which raises the possibility that these results could potentially be generalized among multiple ethnicities. The variation of diets investigated in the studies reviewed is also a strength as it helps to compare different dietary aspects that may result in improved pain symptoms among FMS patients. Another strength is the inclusion of participants aged 12 to 74 years in the studies reviewed. This allows for the generalization of results over a wide age range of individuals with FMS. Eleven of the 12 studies were intervention trials, which allow for more control of potential confounding than observational studies.

There are several limitations in this systematic review. First, most studies did not provide information about the adherence to the diets, which might have affected the study results. Another limitation is that most of the participants were female in all 12 studies, with five studies having only female participants. In the six other studies men only made up 3–22% of the participants, which is not in line with the 3:1 female to male ratio of FMS prevalence. Future studies need to include more men to increase the generalizability to both sexes. An additional limitation is the variety of pain symptom assessments used across all 12 studies and within each of the diet categories. Therefore, it was difficult to make comparisons between studies that did not use the same assessments. One of the assessment types included in this review, TPC, was a major diagnostic criterion from the ACR 1990. The ACR was updated in 2010, some changes being the removal of TPC and the addition of a widespread pain index, as well as a symptom severity scale. For this review it was necessary to include studies that used TPC, as there were not sufficient studies on diet and pain symptoms among FMS patients after 2010. For future studies, the updated pain assessments should be included alongside FIQR, VAS, possibly TPC, TMS, and PPT as well. All 12 studies reviewed had sample sizes below 100, which reduced the power to detect a statistically significant effect of the diets on pain symptoms.

## 5. Conclusions

The results from this systematic literature review suggest that there is potential that diet can be helpful in improving pain symptoms in patients with FMS. From this review, plant-based diets seem to have more consistent and overall success in lessening pain symptoms than elimination/restrictive diets. Given that a limited number of studies have been conducted to date, findings from gluten-free and anti-inflammatory diet studies need to be followed up in future studies. Nevertheless, further studies should be conducted for all four diet categories included in this review and be completed with larger sample sizes and longer intervention periods. Furthermore, using dietary intervention implementation strategies to enhance participants’ adherence to the diet regimen, and including body weight and biomarker measurements to explore potential biological mechanisms are other ways to advance research. In terms of clinical application, there is currently very weak to insufficient evidence for any of the four diet categories to change the status of ‘no recommended diet’ for FMS in the clinical practice.

## Figures and Tables

**Table 1:** Characteristics of studies included in the systematic review.

First Author, Publication Year	Study Design	Year(s)	Country	Number of Participants	Sex ^a^	Age ^b^	Race or Ethnicity	Diagnosis ^c^
Azad 2000 [21]	Intervention Control Trial	No data	Bangladesh	78	Female 78% Male 22%	30.9 (12–60)	No data	FMS
Correa-Rodríguez 2020 [16]	Observational, Cross-sectional	2018	Spain	95	Female 100%	55.76	No data	FMS
Donaldson 2001 [22]	Intervention Pre and Post Trial	No data	United States	20	Female 93% Male 7%	(45–54)	No data	FMS
Hänninen 2000 [27]	Intervention Pre and Post Control Trial	No data	Finland	33	No data	51	No data	FMS
Holton 2012 [23]	Intervention Pre and Post Trial	No data	United States	37	Female 92% Male 8%	51.6	No data	FMS and IBS
Lamb 2011 [18]	Intervention Cross-over Trial	2008	United States	8	Female 100%	55.6 (48–74)	White	FMS
Marum 2016 [19]	Intervention Pre and Post Trial	2015	Portugal	38	Female 100%	51	No data	FMS and GID
Pagliai 2020 [25]	Intervention Cross-over Trial	No data	Italy	20	Female 95% Male 5%	48.95	No data	FMS
Rodrigo 2013 [17]	Intervention Pre and Post Trial	2007–2012	Spain	7	Female 100%	49 (34–68)	White	FMS, IBS and CD
Senna 2012 [26]	Intervention Control Trial	2011	Egypt	83	Female 90% Male 10%	45.6	No data	FMS
Slim 2017 [24]	Intervention Parallel-group Trial	2012–2014	Spain	55	Female 97% Male 3%	HCD: 53 (32–65) GFD: 52 (36–66)	No data	FMS and GS
Vellisca 2014 [20]	Intervention Control Trial	No data	Spain	72	Female 100%	40.98 (24–65)	No data	FMS

^a^ Based on 75 participants at the start of the trial for the Slim study. ^b^ Mean (range) is included; for the Donaldson study, values are from normal data range for women aged 45–54 noted in the publication [22]; for the Hänninen study, average age was estimated based on another publication of the same study [8]; and for the Slim study the median age (range) is included. ^c^ ACR 1990 Criteria for Fibromyalgia Syndrome and ROME III Criteria for Irritable Bowel Syndrome were used except for Marum, Pagliai and Slim studies. Abbreviations used: celiac disease (CD), fibromyalgia syndrome (FMS), gastrointestinal disorder (GID), gluten-free diet (GFD), gluten sensitivity (GS), hypocaloric diet (HCD), and irritable bowel syndrome (IBS).

**Table 2:** Effects of diets on pain symptoms measured by Revised Fibromyalgia Impact Questionnaire (FIQR) and Visual Analogue Scale for Pain (VAS).

First Author of Article	Dietary Variable or Intervention	Length of Intervention	FIQ/FIQR ^c^			VAS ^d^			
						**Baseline**		**End**	
Azad	Vegetarian	6 weeks				5.7 ± 1.8		5.0 ± 1.8 *	
	Control	6 weeks				6.2 ± 1.9		2.3 ± 1.3 *	
			**Cross-sectional**		**Cross-sectional**				
Correa-Rodríguez ^a^	Anti-Inflammatory diet: DII score Quartile 1	N/A	70.5 ± 13.3			7.20 ± 1.64			
	Quartile 2		79.9 ± 10.5			7.91 ± 1.57			
	Quartile 3		69.2 ± 20.1			7.48 ± 2.10			
	Quartile 4		71.9 ± 15.4			7.40 ± 1.14			
			**Baseline**	**2 months**	**End**	**Baseline**	**2 months**	**End**	
Donaldson	Raw Vegetarian	7 months	51.4 ± 14.2	33.6 ± 15.6	27.6 ± 19.0 *				
						**Baseline**	**Middle**	**End**	
Hänninen ^b^	Living Food	3 months				6.0	3.0	3.2 *	
	Control	3 months				5.8	4.8	6.5	
			**Baseline**	**End**		**Baseline**		**End**	
Holton	Dietary Additive Excitotoxin Elimination	4 weeks	58.6	36.4 *		13.1		7.7 *	
			**Baseline**	**End**					
Lamb	Control	4 weeks	46.3 ± 3.4	43.6 ± 5.1					
	Modified Elimination	4 weeks		36.6 ± 8.2					
			**Baseline**	**End**		**Baseline**		**End**	
Marum	Low FODMAP	4 weeks	61.6	47.9 *		6.6		4.9 *	
			**Baseline**	**End**		**Baseline**		**End**	
Rodrigo	Gluten-Free	1 year	74.3 ± 2.9	36.6 ± 4.0 *		8.0 ± 0.5		3.9 ± 1.0 *	
			**Baseline**	**End**					
Senna	Energy-restricted	6 months	54.6 ± 13.1	47 ± 5.1 *					
	Control	6 months	53.2 ± 11.55	51.6 ± 9.4					
			**Baseline**	**End**					
Slim	Gluten-Free	24 weeks	69.5 ± 16.3	60.3 ± 19.6					
	Hypocaloric (Control)	24 weeks	70.4 ± 16.1	61.7 ± 22.2					
			**Baseline**	**End**					
Pagliai	Khorasan Wheat Replacement	8 weeks	54.3	42.06 *					
	Control	8 weeks	54.06	53.87					
						**Baseline**	**1 month**	**2 months**	**End**
Vellisca	Control	3 months				5.63 ± 0.86	5.41 ± 0.73	5.05 ± 0.82	5.31 ± 0.88
	Dietary Additive Excitotoxin Elimination	3 months				5.58 ± 0.91	5.05 ± 0.82	4.88 ± 0.97	5.15 ± 0.95

^a^ Both diet and pain symptoms were measured at the same time due to a cross-sectional study design. ^b^ Values were estimated based on the graph for Hänninen study. ^c^ FIQ 1991 version (FIQ) were used in Donaldson, Lamb and Rodrigo studies. ^d^ VAS scores were reported with a range 0–10 cm, except for a range 0–20 cm for Holton and a range 0–7 cm for Vellisca studies. * Statistically significant (*p* ≤ 0.05).

**Table 3:** Effects of diets on pain symptoms measured by Tender Point Count (TPC) and Total Myalgic Score (TMS).

First Author of Article	Dietary Variable or Intervention	Length of Intervention	TPC		TMS	
			**Baseline**	**End**	**Baseline**	**End**
Azad	Vegetarian	6 weeks	15.7 ± 2.4	14.7 ± 3.6 *	-	-
	Control	6 weeks	16.1 ± 2.3	6.4 ± 3.0 *	-	-
Holton	Dietary Additive Excitotoxin Elimination	4 weeks	16.5	14.0 *	35.2	25.7 *
Rodrigo	Gluten-Free	1 year	16.3 ± 2.4	8.0 ± 1.6 *	-	-
Senna	Energy-restricted	6 months		4.9 ± 0.8	-	
	Control	6 months		5.7 ± 1		

Abbreviations used: Dietary Inflammatory Index (DII), Fibromyalgia Impact Questionnaire (FIQ), fermentable oligo-, di- or mono-saccharides and polyols (FODMAP), Revised Fibromyalgia Impact Questionnaire (FIQR), and Visual Analogue Scale for Pain (VAS) * Statistically significant (*p* ≤ 0.05).

**Table 4:** Effects of diets on pain symptoms measured by Pain Pressure Threshold (PPT).

First Author of Article	Dietary Variable or Intervention	Length of Intervention	PPT																	
			**Occiput**		**Trapezius**		**Zygapophyseal Joint**		**Supraspinatus**		**Second Rib**		**Epicondyle**		**Gluteus**		**Greater Trochanter**		**Knee**	
			**Cross-Sectional**																	
Correa-Rodríguez ^a^	Anti-Inflammatory: DII score Quartile 1 Quartile 2 Quartile 3 Quartile 4	N/A	1.18 ± 0.79		1.37 ± 0.84		1.50 ± 1.12		1.61 ± 1.14		1.09 ± 0.49		1.24 ± 0.74		2.43 ± 1.63		2.39 ± 0.95		2.17 ± 1.00	
			0.94 ± 0.75		0.97 ± 0.65		0.97 ± 0.72		1.31 ± 0.89		0.87 ± 0.52		0.95 ± 0.59		2.12 ± 1.70		2.36 ± 1.47		1.91 ± 1.43	
			0.72 ± 0.44		0.83 ± 0.41		0.90 ± 0.53		1.08 ± 0.52		0.80 ± 0.37		0.85 ± 0.43		1.65 ± 1.32		1.78 ± 0.87		1.05 ± 1.05	
			0.57 ± 0.37 *		0.73 ± 0.45 *		0.80 ± 0.53 *		0.99 ± 0.51		0.67 ± 0.25 *		0.78 ± 0.35		1.44 ± 0.78		1.65 ± 0.72 *		1.20 ± 0.65 *	
			**BL**	**End**	**BL**	**End**	**BL**	**End**	**BL**	**End**	**BL**	**End**	**BL**	**End**	**BL**	**End**	**BL**	**End**	**BL**	**End**
Lamb	Control	4 weeks	0.89	1.35	1.62	2.11	1.11	1.64	1.85	2.19	1.11	1.03	0.94	1.13	2.38	2.73	1.78	2.26	1.41	1.29
	Modified Elimination	4 weeks		1.48		2.33		2.05		2.50		0.93		1.54		2.81		2.41		1.87
			**End**		**End**		**End**		**End**		**End**		**End**		**End**		**End**		**End**	
Senna	Energy-restricted	6 months	4.5 ± 2.9		6 ± 2.3 *		4.7 ± 1.9		4.6 ± 2.1 *		5.8 ± 1.5		4.9 ± 1.8		4.3 ± 1.8 *		4.9 ± 2.1 *		4.2 ± 1.8 **	
	Control		5.1 ± 2.7		7.3 ± 2.4		4.5 ± 1.7		5.9 ± 2.7		6 ± 1.7		4.3 ± 2.2		5.7 ± 2.2		6.2 ± 2.4		6.1 ± 2	

^a^ Both diet and pain symptoms were measured at the same time due to a cross-sectional study design. * Statistically significant (*p* ≤ 0.05); ** Statistically significant *p* ≤ 0.01. Abbreviations used: Baseline (BL), and Dietary Inflammatory Index (DII). Regarding the gluten-free diet, Rodrigo et al. [17] reported statistically significant decreases in FIQ, TPC, and VAS values after one year of the intervention among participants who were diagnosed with both FMS and Irritable Bowel Syndrome (IBS). In the Slim study [24] the gluten-free diet was compared to a control diet (hypocaloric diet) among patients with FMS and gluten sensitivity symptoms. At the end of the 24-week intervention period, the baseline mean FIQR score of 69.5 decreased to 60.3 in the gluten-free diet group. However, this change in FIQR score did not reveal a statistically significant difference from the hypocaloric control group.

## Data Availability

Data sharing not applicable.
